# Balloon Dilatation in the Management of Congenital Obstructive Lesions of the Heart: Review of Author’s Experiences and Observations—Part II

**DOI:** 10.3390/jcdd10070288

**Published:** 2023-07-06

**Authors:** P. Syamasundar Rao

**Affiliations:** Children’s Heart Institute, University of Texas-Houston McGovern Medical School, Children’s Memorial Hermann Hospital, Houston, TX 77030, USA; p.syamasundar.rao@uth.tmc.edu or srao.patnana@yahoo.com; Tel.: +1-713-500-5738; Fax: +1-713-500-5751

**Keywords:** pulmonary stenosis, aortic stenosis, aortic coarctation, balloon valvuloplasty, balloon angioplasty, infundibular stenosis, right ventricular filling, aortic remodeling

## Abstract

While investigating the outcomes of balloon dilatation procedures in patients with congenital obstructive lesions of the heart, several parallel observations were made. The purpose of this review is to present these observations/phenomena/innovations related to balloon dilatation of pulmonary stenosis (PS), aortic stenosis (AS), and aortic coarctation (AC). In subjects who had balloon pulmonary valvuloplasty (BPV), development of infundibular obstruction, electrocardiographic (ECG) changes, changes in right ventricular filling, role of balloon/annulus ratios on the results of BPV, and double balloon vs. single balloon BPV will be reviewed. In patients who had balloon aortic valvuloplasty (BAV), causes of aortic insufficiency and trans-umbilical venous approach for BAV are tackled. In children who had balloon angioplasty (BA) of AC, aortic remodeling and biophysical response after BA of AC are discussed.

## 1. Introduction

In Part I of this series, indications, techniques, and outcomes of balloon pulmonary valvuloplasty (BPV) of pulmonary stenosis (PS); balloon aortic valvuloplasty (BAV) of aortic stenosis (AS); and balloon angioplasty (BA) of aortic coarctation (AC), both native and post-surgical, were reviewed [1]. In this Part II, observations/phenomena/innovations encountered/made by the author during the process of examining the outcome of BPV, BAV and BA of PS, AS, and AC, respectively, will be attempted.

## 2. Pulmonary Stenosis

In this section, development of infundibular obstruction, electrocardiographic (ECG) changes, changes in right ventricular filling following BPV, role of balloon/annulus ratios on the results of BPV, and double balloon vs. single balloon BPV are discussed.

### 2.1. Development of Infundibular Stenosis

**Figure 1:**
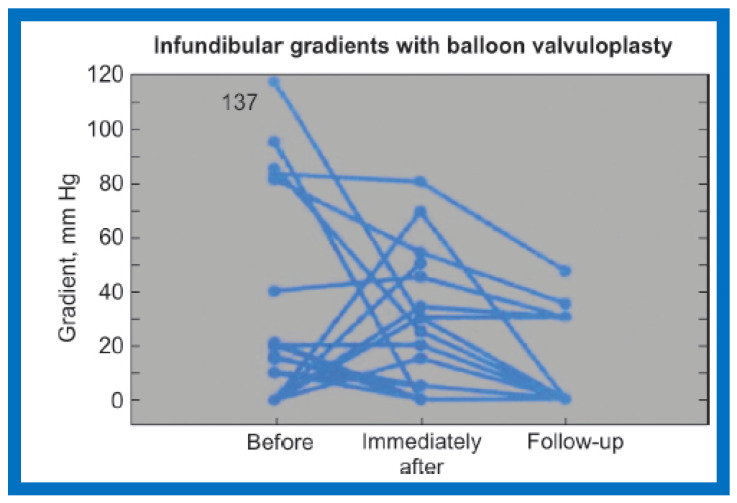
Line graph showing infundibular gradients before and immediately after balloon pulmonary valvuloplasty and at follow-up. Thirteen children had initial gradients; five disappeared immediately after valvuloplasty. New gradients appeared in five other patients. The gradients either improved or disappeared at follow-up. Modified from Reference [2].

After having observed development of right ventricular (RV) infundibular obstruction, we decided to examine the prevalence and significance of infundibular stenosis and its clinical consequences [2]. The information on 62 consecutive patients from ages of seven days to twenty years with a median of six years who had BPV during a 55-month period ending in May 1988 was examined [2]. The peak systolic pressure gradients through the pulmonary valve were reduced (93 ± 43 vs. 27 ± 19 mmHg; *p* < 0.001) at the time of BPV. Systolic pressure gradients across the RV infundibulum were seen in 13 (21%) of the 62 children before BPV. These gradients were 49 ± 42 mmHg and ranged from 10 to 137 mmHg. After BPV, the RV infundibular gradients were eliminated in five children. The gradients persisted in the remaining eight patients; these ranged from 5 to 80 mmHg with a mean of 33 ± 26 mmHg. New RV infundibular gradients emerged in five different children: these varied from 15 to 60 mmHg with a mean of 40 ± 21 mmHg. Figure 1 illustrates RV infundibular gradients observed both pre-BPV and post-BPV as well as at follow-up. 

**Figure 2:**
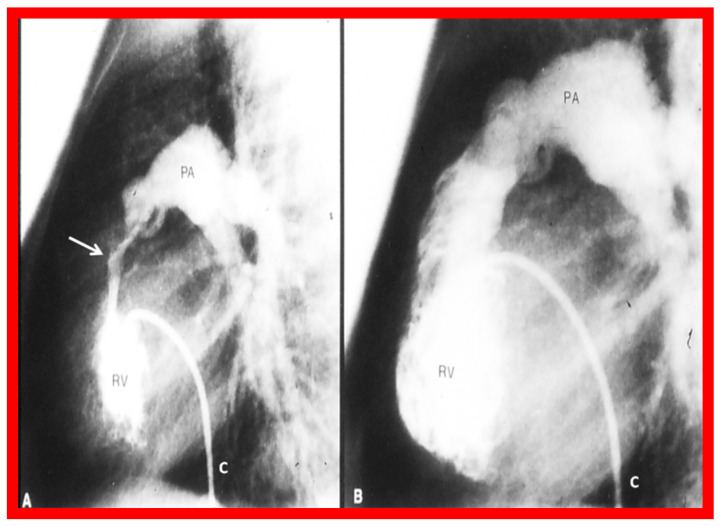
Selected cine frames from right ventricular (RV) angiogram in lateral view, showing severe infundibular stenosis (arrow) (**A**) immediately after balloon pulmonary valvuloplasty. Note the wide-open right ventricular outflow tract (**B**) at cardiac catheterization 10 months after balloon valvuloplasty. The peak-to-peak pulmonary valvar pressure gradient at follow-up catheterization was 20 mmHg; there was no infundibular gradient. C, catheter; PA, pulmonary artery. Reproduced from Reference [4].

**Figure 3:**
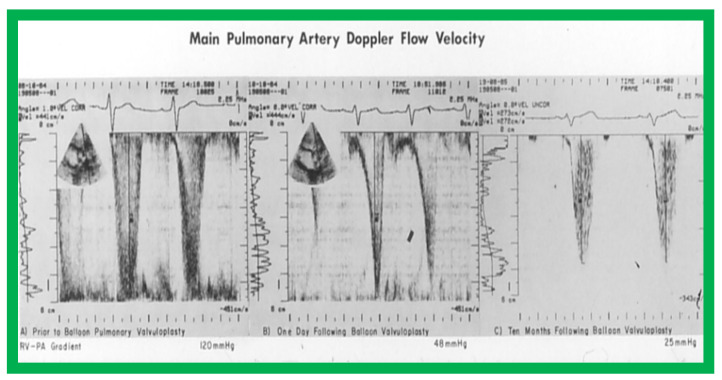
The main pulmonary artery Doppler flow velocities prior to (**A**), and one day (**B**) and ten months (**C**) following balloon pulmonary valvuloplasty are shown. Note that there is a significant fall in the peak flow velocity immediately after valvuloplasty, but a moderate (48 mmHg) gradient that has a characteristic triangular pattern, highly suggestive of infundibular obstruction (corresponding to Figure 2A) persisted. At 10-month follow-up, the flow velocity has markedly diminished, indicating the resolution of the infundibular obstruction (corresponding to Figure 2B). The residual calculated gradients are shown at the bottom of each panel. Reproduced from Reference [4].

Figure 2 and Figure 3 demonstrate instances of RV infundibular stenosis as seen on RV angiogram (Figure 2A) and on Doppler study (Figure 3B (middle panel)). Ten percent children (6 of 62) were administered propranolol either during or following BPV to lessen the RV systolic pressure and to alleviate the RV infundibular stenosis acutely [2,3].

At mid-term follow-up, the RV infundibular stenosis either completely resolved or its magnitude further reduced (Figure 1, Figure 2B and Figure 3C). At the time of the ending of this investigation [2], no patient needed surgery. However, at long-term follow-up [5], three (4%) of 80 patients needed surgery to alleviate fixed RV infundibular obstruction, months, or years after initial BPV. 

**Figure 4:**
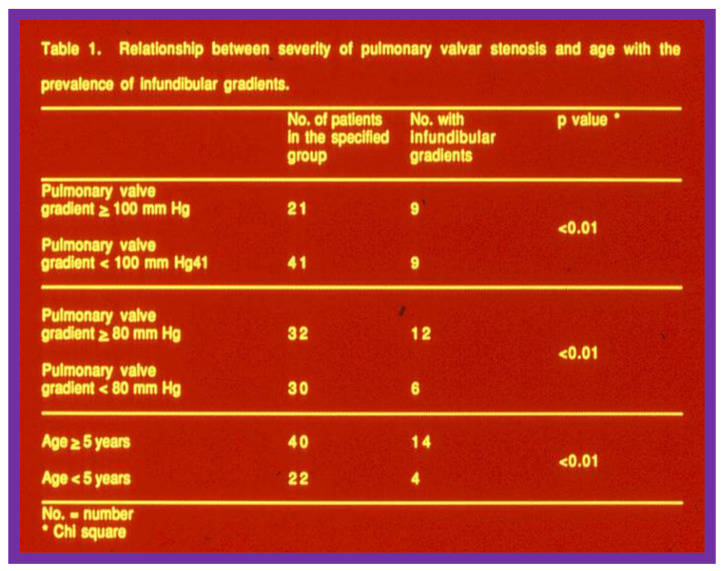
The relationship between the prevalence of infundibular obstruction and age at valvuloplasty and severity of pulmonary valve obstruction. The prevalence of infundibular obstruction is higher with increasing age and increasing degree of pulmonary valve stenosis. Modified from Reference [2].

As reviewed above, 29% of children (18 of 62) were found to have RV infundibular obstruction following BPV. The impact of the age of the patient at BPV and the severity of PS on the occurrence of infundibular stenosis was investigated; the frequency of RV infundibular stenosis was greater with increasing age of the patient and increasing degree of pulmonary valve stenosis (Figure 4) [2].

It should be mentioned that other investigators also examined the phenomenon of RV infundibular stenosis following BPV [6]. Fontes and his associates [6] have examined the importance of RV infundibular obstruction following BPV in patients who had supra-systemic systolic pressures in the RV. These authors examined the outcomes of 33 patients with severe PS; 73% patients developed RV infundibular obstruction after BPV. Their study also demonstrated that the RV infundibular stenosis regresses with time. They recommended administering propranolol in patients with RV infundibular gradients higher than 50 mmHg. Fontes concludes that RV infundibular obstruction is reactive and reversible and suggests that BPV should stay as the therapy of choice in all patients, including severe PS [6]. 

Our observations indicate that RV infundibular gradients occur after BPV in nearly 30% children; a higher prevalence, nearly 75%, is seen in subjects with severe PS with supra-systemic RV pressure [2,6]. The occurrence of infundibular stenosis is more frequent with increasing age and severity of PS. Patients who exhibit systemic or supra-systemic RV pressures due to a hyper-reactive RV infundibulum should be treated with beta blocker drugs [2,6]; such therapy should be instituted if the infundibular gradients are more than 50 mmHg [2,6]. The RV infundibular stenosis seems to regress to a great extent at follow-up (Figure 1, Figure 2 and Figure 3). The possibility for the development of RV infundibular stenosis should not dissuade cardiologists from employing BPV in the treatment of valvar PS. It is recommended that BPV should be undertaken before the age of five years and prior to the developing high (>80 mmHg) pulmonary valve peak systolic gradients to avoid/lessen infundibular stenosis. 

Following an exchange of ideas [7,8], we made recommendations for the diagnosis and management of post-BPV infundibular stenosis [8]; these are summarized in Table 1.

### 2.2. Electrocardiographic Changes following BPV

**Figure 5:**
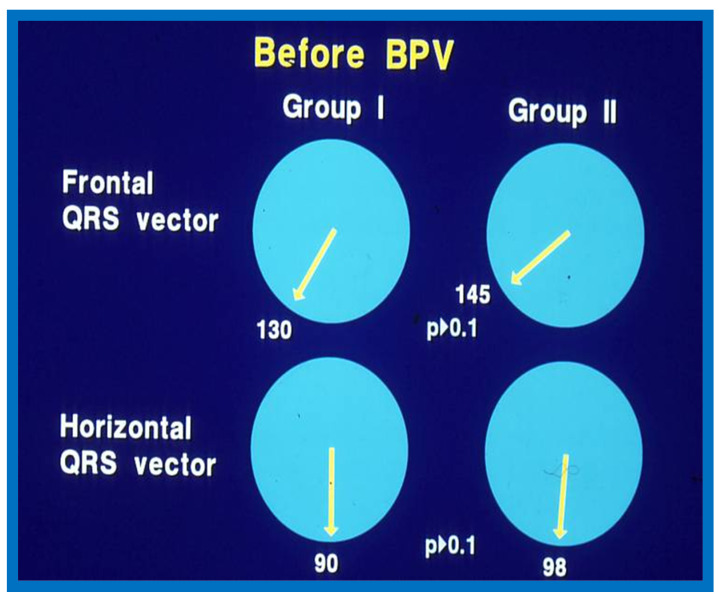
Plots of mean QRS vectors (axis) in the frontal (top) and horizontal (bottom) planes in group I (with good results) (left circles) and group II (with poor results) (right circles) prior to balloon pulmonary valvuloplasty (BPV) are shown. Note that no significant (*p* > 0.1) difference was seen between groups I and II. Reproduced from Reference [20].

**Figure 6:**
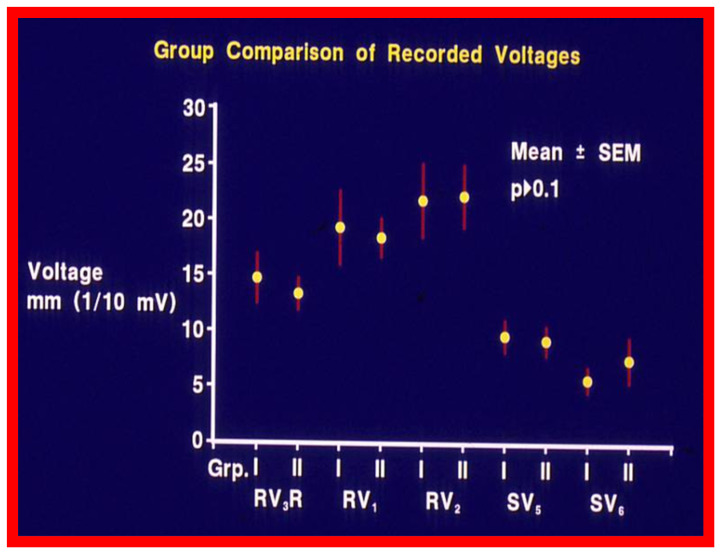
Anterior (R waves in leads V_3_R, V_1_, and V_2_) and terminal rightward (S waves in leads V_5_ and V_6_) voltages in the electrocardiograms prior to balloon pulmonary valvuloplasty are compared between groups (Grp.) I (with good results) and group II (with poor results). Mean and standard error of mean (SEM) are shown. Note that no significant (*p* > 0.1) difference is shown between groups I and II. Reproduced from Reference [20].

While cardiac catheterization-measured pulmonary valve gradients [17] and Doppler peak instantaneous gradients [4,5,18] have been the mainstay in the evaluation of the results of BPV, we sought to examine if the much simpler and routinely performed ECGs have any useful value in assessing the results of BPV. We have analyzed ECGs of 35 patients who underwent BPV to relieve valvar PS to record changes in the ECG following BPV and to assess if such changes indicate reduction in pressure gradients across the pulmonary valve following BPV [19]. The follow-up (at a mean of 11 months after BPV) data of 35 patients were evaluated. They were split into two groups: Group I (N = 30) who had good outcomes, defined as pulmonary valve gradients < 50 mmHg at follow-up and Group II (N = 5) who had poor outcomes, defined as pulmonary valve with gradients ≥ 50 mmHg. Examination of frontal and horizontal plane mean QRS axis (vector) (Figure 5), R waves in leads V_3_R,V_1_, and V_2,_ reflecting anterior RV voltages and S waves in leads V_5_ and V_6,_ reflecting rightward RV voltages (Figure 6) and direction of T waves in the right chest leads were similar (*p* > 0.1) in both Groups I and II before BPV [19]. 

**Figure 7:**
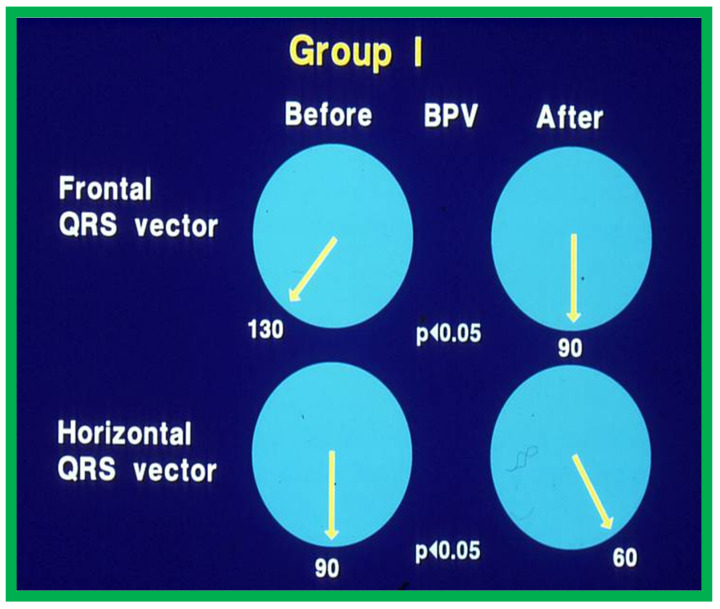
Plots of mean QRS vectors (axis) in the frontal (top) and horizontal (bottom) planes in group I (with good results) prior to balloon pulmonary valvuloplasty (BPV) (left circles) and at follow-up (right circles) are shown. Note the significant (*p* < 0.05) improvement at follow-up. Reproduced from Reference [20].

**Figure 8:**
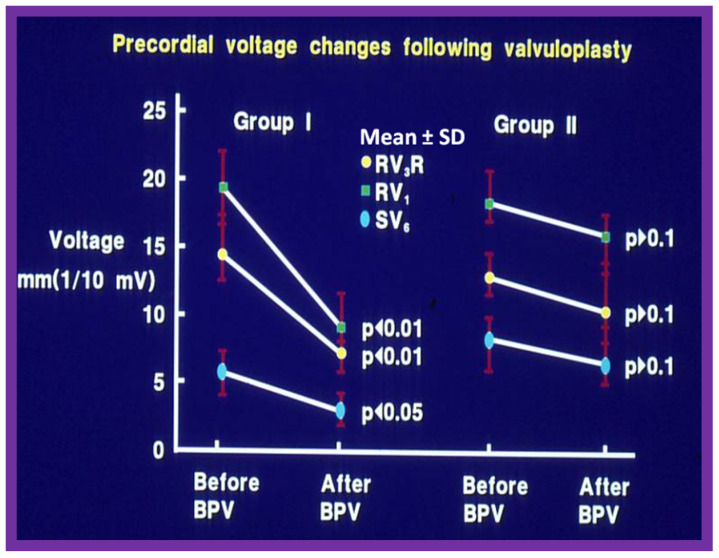
Precordial ECG voltages (R waves in leads V_3_R and V_1_ and S waves in V_6_) prior to and at follow-up after balloon pulmonary valvuloplasty (BPV) in group I (with good results) (left panel) and group II (with poor results) (right panel) are depicted. The mean and standard deviation (SD) are shown. Note the significant (*p* < 0.05 to <0.01) decrease in the voltages in group I while there was no significant (*p* > 0.1) change in group II. Reproduced from Reference [20].

In Group I patients, the frontal plane mean QRS vector moved from 127° ± 25° to 81° ± 47°; *p* < 0.05 and horizontal plane mean QRS vector moved leftward from 88° ± 36° to 27° ± 51°; *p* < 0.05 (Figure 7) at follow-up of after BPV. Similarly, the anterior and rightward RV voltages diminished significantly (*p* < 0.001) (Figure 8, left panel) and the upright T waves became inverted in the Group I patients with good outcomes. 

By contrast, in Group II patients with poor results, no change in frontal (145° ± 27° vs. 145° ± 27°) and horizontal (98° ± 19° vs. 112° ± 29°) plane mean vectors occurred. Similarly, the anterior and rightward RV voltages did not change (*p* > 0.1) as shown in Figure 8, right panel. In addition, upright T waves in right chest leads did not return to normal in poor results group. 

**Figure 9:**
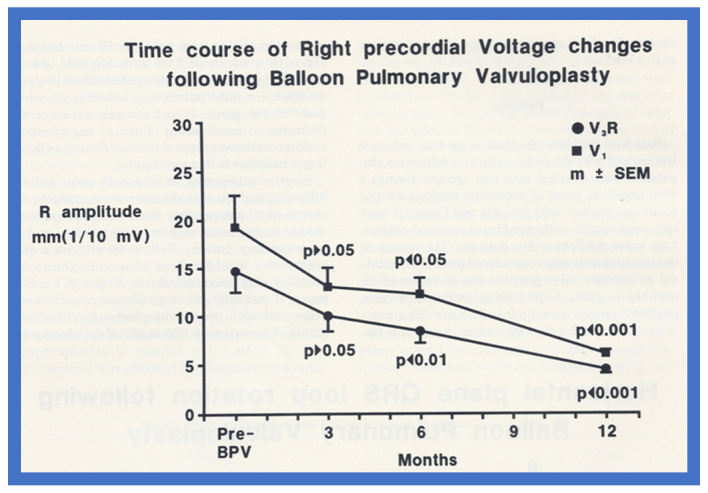
Precordial ECG voltages (R waves in leads V_3_R [circles] and V_1_ [squares]) prior to and at 3, 6, and 12 months following balloon pulmonary valvuloplasty (BPV) in group I patients (with good results). Note that a gradual improvement was shown; at 3-month follow-up, there was no statistically significant decrease (*p* > 0.05), but at 6 and 12 months, a significant (*p* < 0.05 to 0.001) decrease was observed. The mean and standard error of mean (SEM) are shown. Reproduced from Reference [19].

**Figure 10:**
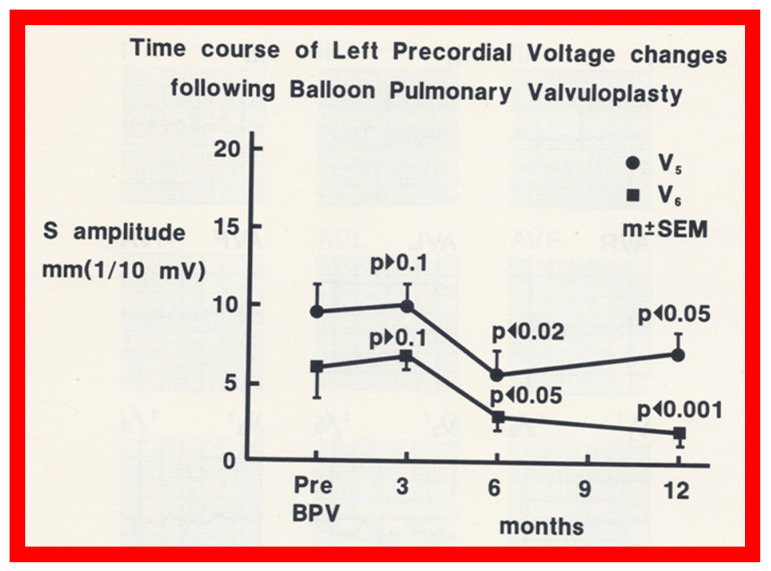
Precordial ECG voltages (S waves in leads V_5_ (circles) and V_6_ (squares)) prior to and at 3, 6, and 12 months following balloon pulmonary valvuloplasty (BPV) in group I patients (with good results). Note that a gradual improvement was shown; at 3-month follow-up, there was no statistically significant decrease (*p* > 0.1), but at 6 and 12 months, a significant (*p* < 0.02 to 0.001) decrease was observed. The mean and standard error of mean (SEM) are shown. Reproduced from Reference [19].

After having demonstrated that the RV hypertrophy seen on ECG regresses at follow-up after successful BPV (Group I), we attempted to investigate time course of changes in the RV voltages following BPV. There was no reduction (*p* > 0.05) in the RV voltages at 3 months after BPV (Figure 9 and Figure 10). However, at 6 and 12 months after successful BPV the RV voltages were reduced (*p* < 0.05 to <0.001) (Figure 9 and Figure 10).

**Figure 11:**
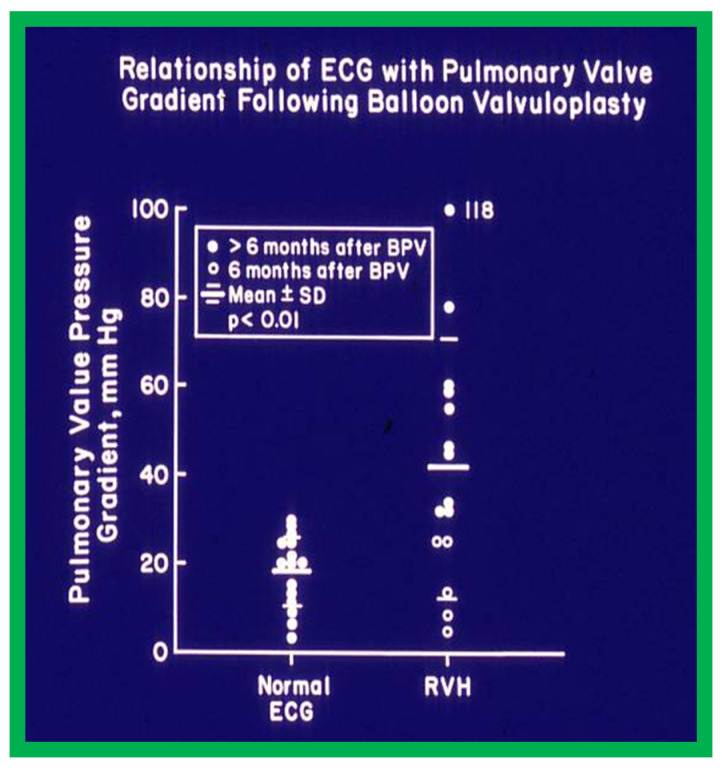
The relationship of residual pulmonary valve gradients at follow-up after balloon pulmonary valvuloplasty (BPV) and electrocardiogram (ECG) is plotted. Note that a normal ECG is found in patients with minimal residual pulmonary valve gradients (left panel) while RVH indicates a significant residual gradient, or that the ECGs were recorded earlier than six months after BPV. The mean and standard deviation (SD) are shown. Filled circles—ECGs recorded six months after BPV. Open circles—ECGs recorded prior to six months after BPV. ECGs recorded prior to six months after BPV exhibited RVH, despite reduced gradients; this may in part be related to not yet having had a chance for the complete resolution of RVH. Reproduced from Reference [20].

We then investigated to see if the post-BPV ECG can predict pulmonary valve residual gradient. Examination of 30 pairs of ECG and catheterization-measured gradients across the pulmonary valve obtained within 24 h of each other discovered that pulmonary valve gradients less than 30 mmHg are likely to be seen in patients with normal ECG (Figure 11). If RV hypertrophy is found in the ECG obtained at or later than 6 months following BPV, significant residual gradient is expected (Figure 11). However, RV hypertrophy in the ECG secured prior to 6 months after BPV does not correctly foresee residual gradient across the pulmonary valve.

Based on these data, it was concluded that ECG gets better after successful BPV, and the ECG is a valuable adjunct in the evaluation of outcomes of BPV. ECG confirmation of improved gradient across the pulmonary valve does not become evident until six months following BPV. 

Other investigators also examined ECG changes following BPV; leftward shift of frontal plane mean vector [21,22], decrease in RV voltages [21,22], reversion of upright T waves lead V_1_ [23], and regression of ECG signs of RV hypertrophy [21,22,23] have been found; these observations are similar those of ours [19]. 

While echo-Doppler studies provide direct evidence for relief of pulmonary valve obstruction, ECG, which is performed at most institutions as part of routine follow-up evaluation, may serve as an adjunct. 

### 2.3. Changes in Right Ventricular Filling

**Figure 12:**
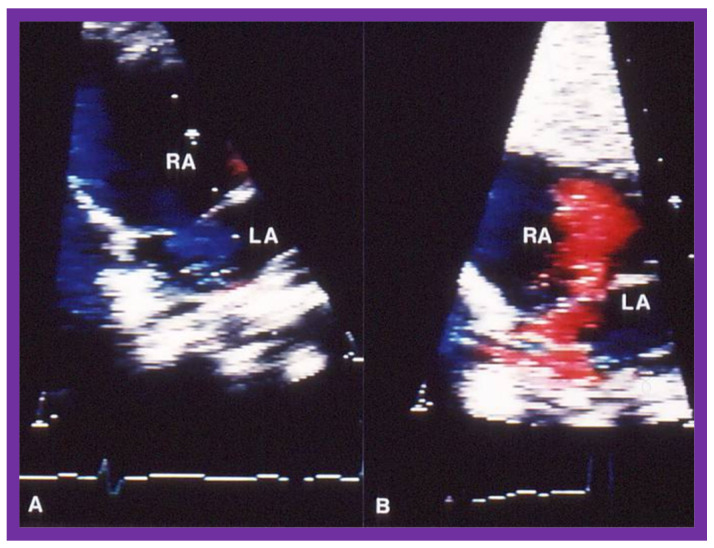
Selected video frames of the atrial septum, demonstrating a right-to-left shunt by color Doppler, across the patent foramen ovale prior to balloon pulmonary valvuloplasty (**A**) which has changed to a left-to-right shunt (**B)** 24 h later. LA, left atrium; RA, right atrium. Reproduced from Reference [20].

**Figure 13:**
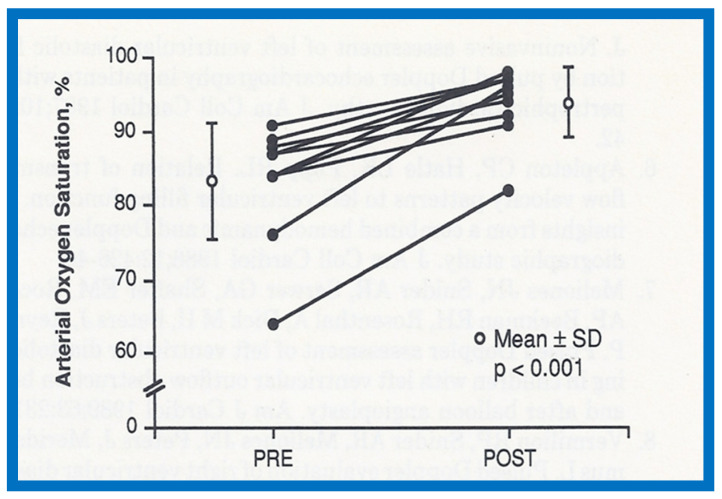
Arterial oxygen saturations prior to (PRE) and 15 min after (POST) balloon pulmonary valvuloplasty in each of the 10 patients (solid circles) are shown. There is an increase in saturation in all patients. The mean (open circles) ± standard deviation (SD) is also shown. There is a statistically significant (*p* < 0.001) increase in oxygen saturation. Reproduced from Reference [27].

Abnormalities of RV filling and reduced compliance of the RV musculature are anticipated in children with RV outflow tract obstruction in a manner like those seen in patients with left ventricular outflow obstruction [24,25]. Similar abnormalities were shown in patients with RV hypertrophy associated with PS [26]. However, Vermilion and associates [26] could not demonstrate any change in the RV filling abnormalities and compliance parameters after successful BPV [26]. Because these findings are at variance with our clinical observations, we examined this issue with the aid data from our patients [27]. Of a total of 75 patients who had BPV, 10 patients had arterial desaturation due to right to left shunt through the patent foramen ovale (PFO) (Figure 12A). In this subset of patients, the peak pulmonary valve gradients decreased (118 ± 38 mmHg vs. 28 ± 17 mmHg; *p* < 0.001) immediately after BPV. The right to left shunt across the PFO was reversed (Figure 12B). Simultaneously, the systemic arterial O_2_ saturations improved from 83 ± 8% to 94 ± 5% (*p* < 0.001), the pulmonary flow index increased from 1.9 ± 0.5 to 2.7 ± 0.6 L/min/m^2^ (*p* < 0.001), and the pulmonary to systemic flow ratio was elevated from 0.7 ± 0.1 to 0.95 ± 0.1 (*p* < 0.001). When the information on each child was examined, the arterial O_2_ saturation improved in every child (Figure 13) [27].

Improved O_2_ saturation after BPV is linked to eliminated or diminished right to left shunt across the PFO. The only change between pre- and post- BPV status is reduced gradients across the pulmonary valve with consequent reduction in RV afterload. This reduced RV afterload, we believe, has improved RV filling with resultant decrease in right to left atrial shunt [27]. Conceivably the Doppler data [26] are not sufficiently sensitive to perceive improved RV filling following BPV.

### 2.4. Role of Balloon/Annulus Ratios on the Results of BPV

After having examined the influence of size of the balloons used for BPV in a limited number (N = 22) of patients [28], the author sought to explore the role of balloon/annulus (B/A) ratios in a larger group of patients [15,16]. The results of 68 BPV procedures in 56 patients performed during a 50-month period ending in December 1987 were reviewed. The patients’ ages ranged between seven days and 20 years. Initially, the patients were separated into two groups: Group I. Subjects who had BPV with balloons resulting in B/A ratios ≤ 1.0 and Group II. BA ratios > 1.0. 

**Figure 14:**
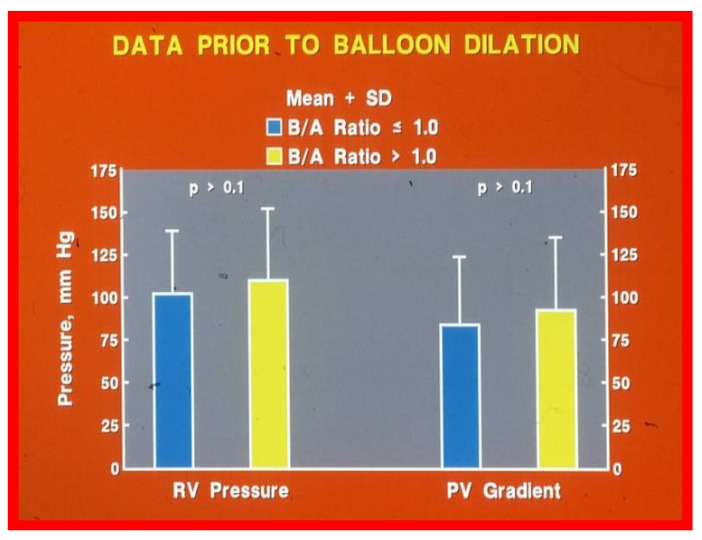
Bar graph demonstrating similar (*p* > 0.1) right ventricular (RV) peak pressures and pulmonary valve (PV) gradients prior to balloon valvuloplasty in Group I with balloon/annulus (B/A) ratio ≤ 1.0 and Group II with balloon/annulus (B/A) ratio > 1.0. Mean + standard deviation (SD) is shown.

Group I consisted of 12 BPVs with B/A ratios ranging between 0.76 and 1.0 (mean = 0.8) and Group II had 56 BPVs with B/A ratios ranging between 1.01 to 1.8 (mean = 1.31). The RV systolic pressures (Figure 14, left column) and peak systolic pressure gradients across the pulmonary valve (Group I—84.3 ± 39.2 mmHg; Group II—92.8 ± 42.1 mmHg; *p* > 0.1; Figure 14, right column) were similar prior to BPV. 

**Figure 15:**
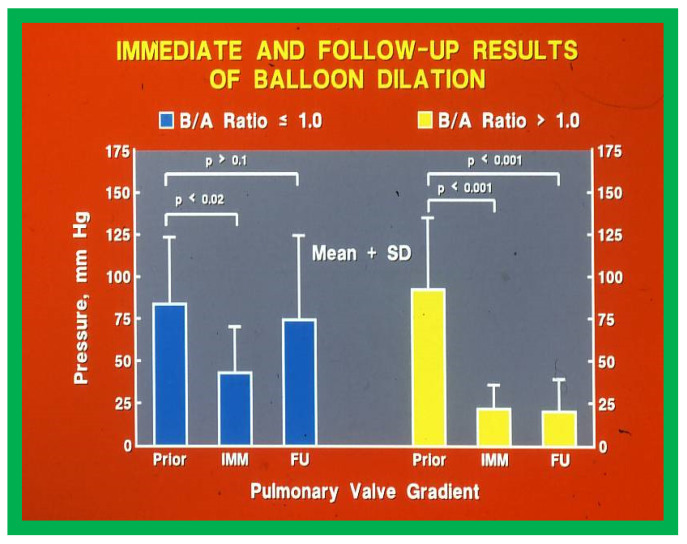
The peak pressure gradient across the pulmonary valve fell immediately (IMM) after balloon dilatation in both Group I with small balloons (*p* < 0.02) and Group 2 with large balloons (*p* < 0001). On intermediate term follow up (FU), the gradient had risen towards pre-dilatation values (*p* > 0.1) in Group I patients (treated with balloons that were smaller than the pulmonary valve annulus) while in Group 2 (balloons larger than the pulmonary valve annulus) the gradients remained low.

There was a significant reduction in gradients immediately after BPV in both groups (Group I: 84 ± 39.2 mmHg vs. 43.6 ± 26.8 mmHg; *p* < 0.02 and Group II: 92.8 ± 42.1 mmHg vs. 22.4 ± 13.6; *p* < 0.01) (Figure 15). However, the residual gradients across the pulmonary valve in Group II patients with large balloons were lower (*p* < 0.01) than those in Group I subjects with small balloons. At intermediate term follow up (6–34 months), the gradient across the pulmonary valve increased to 75.0 ± 49.4 mmHg in Group I (small balloons) and were similar to pre-BPV values (*p* > 0.1) (Figure 15, left panel) while in Group II (large balloons) the gradient across the pulmonary valve stayed lower (20.8 ± 18.5 mmHg; *p* < 0.001) and was not different (*p* > 0.1) from the valve gradients measured immediately following BPV (Figure 15, right panel). Four patients in Group I and one child in Group II required repeat BPV (*p* < 0.05) at follow up.

The above presented data indicate that while the immediate results with both small and large balloons are satisfactory, balloons larger than the pulmonary valve annulus (B/A ratio > 1.0) result in providing lasting relief of pulmonary valve obstruction. 

**Figure 16:**
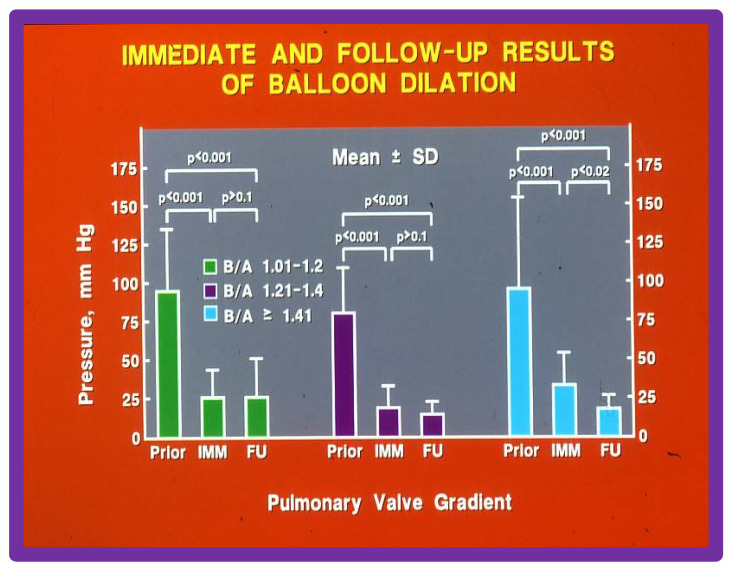
Comparison of immediate (IMM) and follow-up (FU) results of BPV with varying balloon sizes; B/A ratios of 1.0 to 1.2 (left panel), 1.2 to 1.4 (middle panel), and ≥ 1.41 (right panel) had equally good IMM and FU results in terms of reduction in PV gradient (*p* < 0.001). Consequently, use of balloons larger than those resulting in B/A ratios greater than 1.4 have no advantage beyond what is provided by B/A ratios between 1.0 and 1.4. Prior, before balloon valvuloplasty; IMM, immediate; FU, follow-up; BPV, balloon pulmonary valvuloplasty; B/A, balloon-to-annulus; SD, standard deviation; PV, pulmonary valve. Modified from Reference [20].

We then further sub-divided Group II into Subgroups IIA. B/A ratio of 1.0 to 1.2, IIB. B/A ratio of 1.21 to 1.4, IIC. B/A ratio > 1.41, and IID. B/A ratio > 1.5. The gradients across the pulmonary valve were similar (*p* > 0 1) in subgroups IIA, IIB and IIC (Figure 16). The gradients across the pulmonary valve were reduced immediately after BPV and remained low at intermediate-term follow-up in all the subgroups (Figure 16). In subgroup IIC in whom we used the largest B/A ratio (>1.41) there was an additional reduction (*p* < 0 02) gradients at follow up. 

The number of children with residual pulmonary gradients > 30 mm Hg and the number of children needing repeat BPV during follow-up were scrutinized (Table 2). Group IIA subjects with B/A ratio of 1.01 to 1.2 had residual pulmonary valve stenosis whereas Group IIB with B/A ratio of 1.21 to 1.4 and Group IIC with B/A ratio > 1.41 had no residual PS (*p* < 0.005). Group I patients with B/A ratio < 1.0 had the worst outcomes (Table 2). 

**Figure 17:**
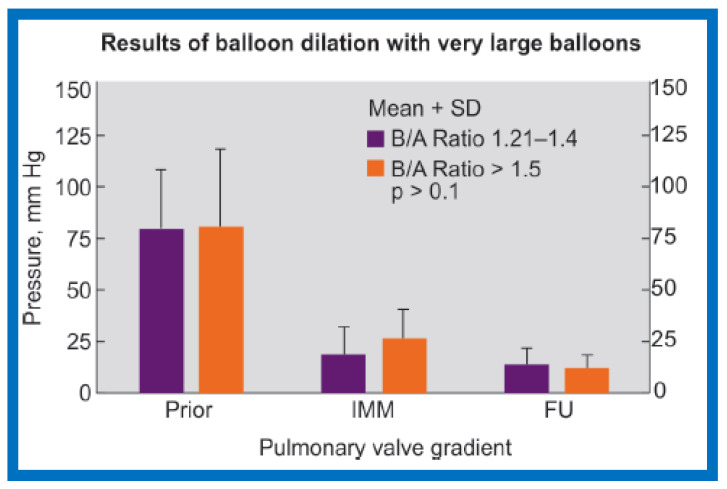
Bar graph shows PV gradients before (prior to) (left panel), immediately after (IMM) (middle panel), and at FU (right panel). There is no statistically significant difference (*p* > 0.1) between the use of B/A ratios of 1.2–1.4 and (≥1.5), implying that large balloons (B/A) ratios (≥1.5) have no advantage beyond what is provided B/A ratios of 1.2–1.4. PV, pulmonary valve; FU, follow-up; B/A, balloon-to-annulus; SD, standard deviation. Reproduced from Reference [20].

Lastly, the outcomes of BPV of children with B/A ratios > 1–5 (Group IID, nine children) were compared with those of Group IIB (B/A ratio of 1.21 to 1.4) to investigate whether there is any benefit in utilizing the oversized balloons (Figure 17). The RV peak systolic pressures (98.0 ± 27.1 vs. 99.0 ± 36.9 mmHg; *p* > 0 1) and gradients across the pulmonary valve (80.7 ± 28.6 vs. 82.1 ± 37.4 mmHg; *p* > 01) (Figure 17, left column) were comparable in both these subgroups. Residual peak pressure gradients across the pulmonary valve immediately following BPV (19.2 ± 13.3 vs. 27.8 ± 13.3; *p* > 0 1) (Figure 17, middle column) and on follow-up (14.9 ± 7.7 vs. 14.0 ± 5.6; *p* >0 1) (Figure 17, right column) were also alike. None of the children in either of these subgroups required repeat BPV nor any child had residual gradient across pulmonary valve greater than 30 mmHg. Furthermore, there was no angiographic suggestion of asymptomatic tears of the pulmonary arterial wall in either subgroup.

This data would indicate that B/A ratios > 1.5 have no added benefit over the subgroup with B/A ratios of 1.21 to 1.4. In addition, reports of damage to the RV outflow tract with the use of balloons > 1.5 times pulmonary valve annulus [14] suggest that balloons that give a B/A ratio of 1.21 to 1.4 are the best for relieving the pulmonary valve obstruction [15,16]. The B/A ratio of 1.21 to 1.4 were used for the next decade or so by most cardiologists. In 1999, Berman and his colleagues [29] reported occurrence of severe pulmonary insufficiency (PI) following BPV, some requiring pulmonary valve replacement. Revisiting of B/A ratios with the objective of eliminating/reducing pulmonary insufficiency resulted in recommendation of B/A ratios of 1.2 to 1.25 as optimal for BPV [9].

The efficacy of B/A ratio of 1.21 to 1.4 in effectively decreasing gradients across the pulmonary valve acutely [30] and both acutely and at follow-up [15,16,28] is well recognized. Because of emergence of PI as a late complication, smaller balloons with B/A ratios of 1.2 to 1.25 were recommended [9]. Such smaller balloons are likely to result in effective relief of PS while at the same time may aid in avoiding significant PI at long-term follow-up [9,10,31,32,33].

### 2.5. Double Balloon vs. Single Balloon BPV

**Figure 18:**
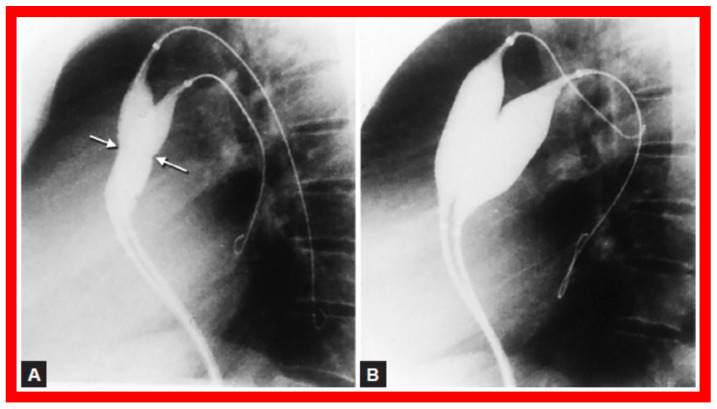
Selected cineradiographic frames in lateral view demonstrating two balloon catheters placed across the PV showing “waisting” of the balloons (arrows) during the initial phases of balloon inflations (**A**) which was completely abolished after complete inflation of balloons (**B**) PV, pulmonary valve. Reproduced from Reference [17].

**Figure 19:**
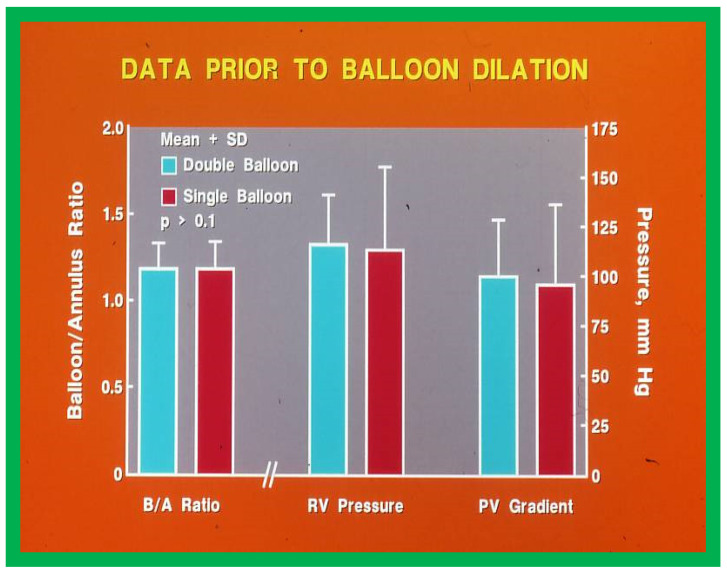
Bar graph comparing balloon/annulus (B/A) ratios of double balloon group with single balloon group. Note that the B/A ratios and right ventricular (RV) pressures and pulmonary valve (PV) gradients prior to balloon valvuloplasty are similar (*p* > 0.1). Mean + standard deviation is marked. Modified from Reference [11].

Double-balloon technique (Figure 18) instead of single balloon usage for BPV was recommended by some cardiologists, specifically for adult patients [34]. We compared the outcomes of single with double-balloon BPV [11] to see if such a recommendation is valid in children with PS. Fifty-four patients aged 7 days to 20 years underwent BPV during a 50-month period ending December 1987. From among this patient population, results of twelve patients who had BPV with two balloons simultaneously placed across the pulmonary valve (Group I) were compared with the outcomes of twelve patients who had BPV with single balloon technique (Group II). Both groups were similar regarding B/A ratios used for BPV (1.19 ± 0.14 vs. 1.19 ± 0.15; *p* > 0.1) and pre-BPV RV peak systolic pressure (116.6 ± 24.5 vs. 113 ± 41.5 mmHg; *p* > 0.1) and the peak systolic pressure gradients across the pulmonary valve (100.5 ± 28.0 vs. 96.3 ± 40.1 mmHg; *p* > 0.1) (Figure 19).

**Figure 20:**
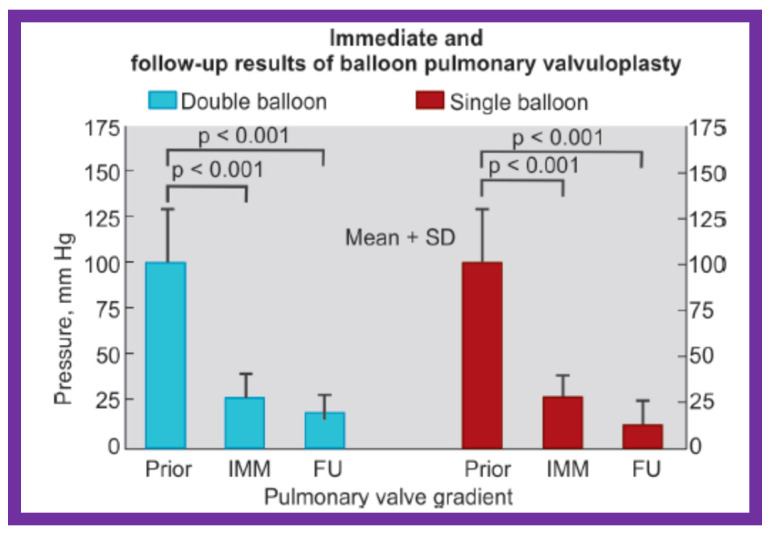
Bar graph showing immediate (IMM) and follow-up (FU) results of BPV using double (left panel) and single (right panel) balloon techniques with equivalent-sized B/A ratios. Note equally significant (*p* < 0.001) reduction in PV gradients both immediately and at FU. The degree of reduction is similar (*p* > 0.1) in both groups. Mean + SD are shown. Prior, before valvuloplasty; BPV, balloon pulmonary valvuloplasty; B/A, balloon-to-annulus; PV, pulmonary valve; SD, standard deviation. Modified from Reference [11].

**Figure 21:**
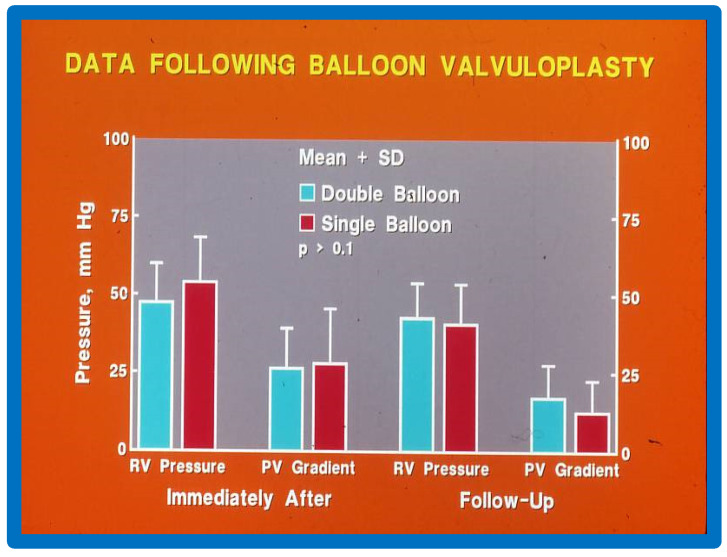
Bar graph showing RV pressure and PV gradients immediately (IMM) after and at follow-up (FU) after BPV with double- and single-balloon techniques with equivalent-sized balloon/annulus ratios. Note no significant difference (*p* > 0.1) between double- and single-balloon techniques. Mean + SD are shown. RV, right ventricular; PV, pulmonary valve; BPV, balloon pulmonary valvuloplasty; SD, standard deviation. Modified from Reference [11].

Immediately following BPV, the peak systolic pressure gradients across the pulmonary valve decreased from 100.5 ± 28.0 to 26.6 ± 12.5 mmHg (*p* < 0.001) in Group I patients with double balloon BPV (Figure 20, left panel). The gradient reduction in Group II patients with single balloon BPV was 96.3 ± 40.1 vs. 28.3 ± 17.3 mmHg (*p* < 0.001) (Figure 20, right panel) and was similar (*p* > 0.1) to double balloon technique (Figure 20). At intermediate-term follow-up, the residual pulmonary valve gradients decreased further in both groups (Group I—17.5 ± 10.2 mmHg and Group II—12.8 ± 9.9 mmHg) and these values were also similar (*p* > 0.1) (Figure 20). Figure 21 illustrates comparison RV systolic pressures and peak pulmonary valve gradients both immediately after BPV and at follow-up; these values are similar (*p* > 0.1) for both groups. Mild PI was seen in seven patients in Group I and eight patients in group II (*p* > 01). None of the patients in either group required repeat BPV or surgery and neither group had any patient with peak pulmonary gradients more than 30 mmHg.

Thus, this study revealed that the results of both procedures were good and similar to each other (Figure 20 and Figure 21). This is subject to similar B/A ratios [11,35]. Furthermore, the double-balloon method prolongs the procedure and needs a second femoral venous entry. Additionally, large diameter balloon catheters are presently accessible and therefore, it is possible to utilize single balloon BPV without the need for double balloon technique. Nevertheless, the double-balloon technique may be more useful in achieving stable balloon position across the pulmonary valve in some adolescent and adult patients.

## 3. Aortic Stenosis

In this segment, causes of aortic insufficiency and trans-umbilical venous approach for BAV will be tackled.

### 3.1. Causes of Aortic Insufficiency

**Figure 22:**
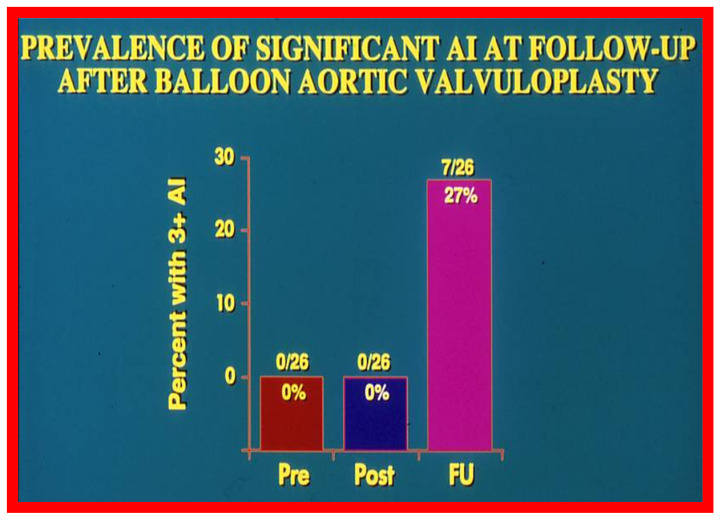
Bar graph demonstrating the prevalence of grade III aortic insufficiency prior to (Pre), immediately following (Post) balloon aortic valvuloplasty and at late follow-up (FU). Note significant increase at late follow-up. Modified from Reference [37].

**Figure 23:**
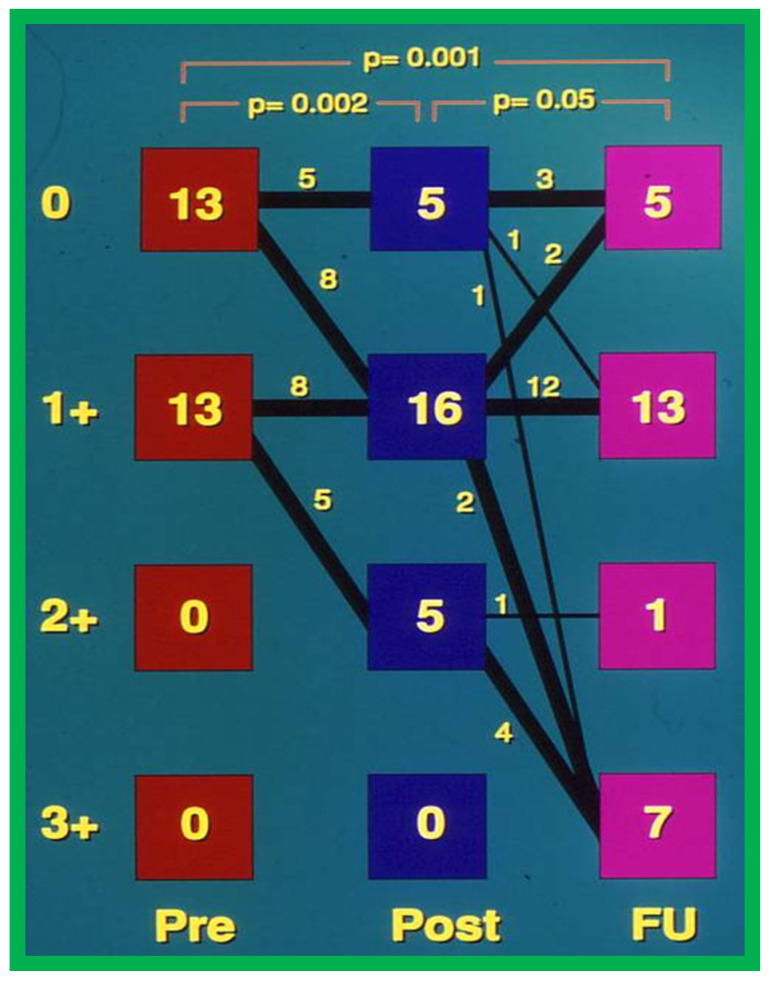
Degree of aortic insufficiency by Doppler echocardiography before (Pre), the day after (Post), and at late follow-up (FU). There is a significant (*p* = 0.002) increase in aortic insufficiency from pre-valvuloplasty to post-valvuloplasty. None were grade 3+ aortic insufficiency. Number of patients with grade 3+ aortic insufficiency (0 of 26 vs. 7 of 26) at follow-up (FU) increased (*p* < 0.02). Modified from Reference [36].

Significant aortic insufficiency (AI) was detected at long-term follow-up of BAV patients [36] as illustrated in Figure 22 and Figure 23. Many other studies demonstrated an inclination for increase in the degree of AI with time; longer the follow up, the greater the AI; significant AI was found in 24 to 38% patients as shown in tabular form elsewhere [37].

**Figure 24:**
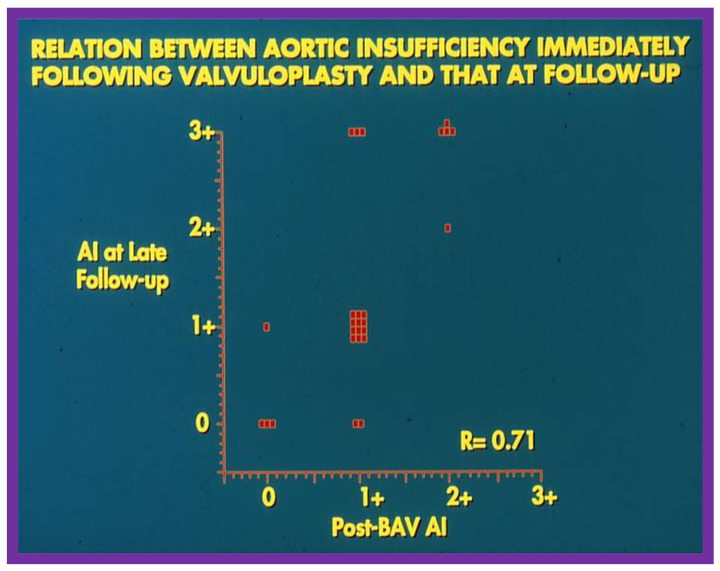
Relationship of immediate post-valvuloplasty Doppler-estimated aortic insufficiency (AI) with AI at late follow-up after balloon aortic valvuloplasty (BAV). Note good correlation (R = 0.71) between the two. Modified from Reference [36].

We sought to examine causes for development of AI [36] at follow-up. In this study, the patients were divided into two groups: Group I. Nineteen children with no significant AI (grade 2+ or less) and Group II. Seven children with 3+ AI. Fifteen anatomic, physiologic, biographic, and procedural data (Table II of Ref. [36]) were scrutinized by multivariate logistic regression assessment to detect factors causing AI [36]. This examination detected three items that were statistically different between the two groups (Table IV of Ref. [36]). These factors are AI magnitude by Doppler both before and immediately after BAV and BAV performed in the latter half of the study. These three items were entered into a multivariate logistic regression model with all likely combinations. A model that involves Doppler-quantitated AI immediately after BAV fits the data best. There was no additional advantage by including pre-BAV Doppler AI and procedural experience to the model that includes immediate post-BAV AI by Doppler, and such a procedure did not enhance its predictive capability [36]. Consequently, we concluded that immediate post-BAV degree of AI is predictive of late development of significant AI. The correlation between these two factors is shown in Figure 24. 

**Figure 25:**
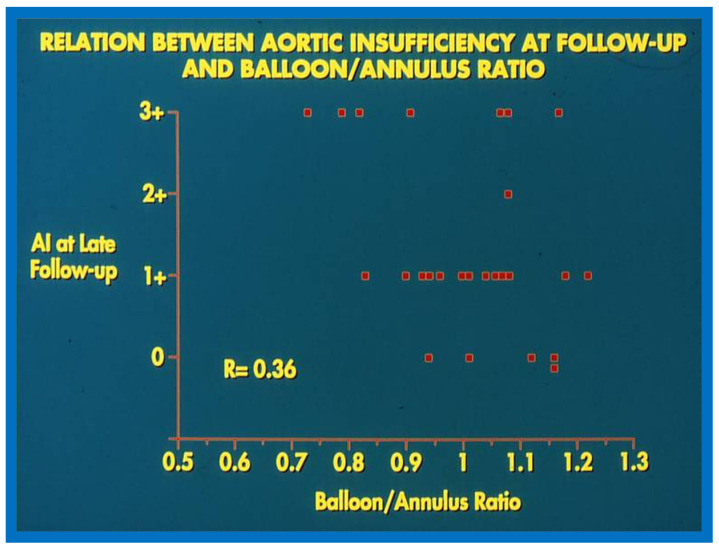
Relationship of balloon/annulus ratio utilized during balloon aortic valvuloplasty with the degree of Doppler-assessed aortic insufficiency (AI) at late follow-up. Note poor correlation (R = 0.36) between these two parameters. Also note grade 3+ AI occurred with wide range of balloon/annulus ratios. Modified from Reference [36].

Balloons larger than 1.2 to 1.5 times the aortic valve annulus have been shown to cause injury to the aortic valve, including aortic valve leaflet tears both in animal models [38] and in human subjects [39] and produce AI. So, we compared the magnitude of AI at late follow-up with the B/A ratios used during BAV and could not establish any correlation between the B/A ratios and the level of AI (Figure 25). 

The causes for development of severe AI at long-term follow-up of BAV are not clearly known. Several hypotheses have been put forward by investigators working on this issue. These are better pressure gradient relief at the time of BAV [38], AI (quantitated by Doppler) both pre-BAV and immediate post-BAV [38], aortic valves that are uni-commissural [40], prolapse of the aortic valve [41], poor valve morphology [36], and large B/A ratio [40,41,42]. However, there does not seem to be any evidence to support that any of these factors are solely responsible for causing AI at late follow-up. Our study [36] suggested that the level of AI at the time of BAV foresees the development of substantial late AI (Figure 24). We also speculated that a mixture of poor valve morphology and use of large-sized balloons are likely to become causative factors for AI at long-term follow-up [36,37,43,44]. Further investigations to study the afore mentioned and other causes of late AI and develop techniques to avoid AI were urged [36,37].

### 3.2. Trans-Umbilical Venous Approach for BAV

While percutaneous femoral arterial route is the most frequently used method for executing BAV, there is a concern for injury to the femoral artery [45,46], chiefly in neonates and young infants. Consequently, other approaches, namely, carotid artery [47], axillary artery [48], umbilical artery [49], subscapular artery [50], anterograde femoral vein [51,52], and umbilical vein [53,54] routes have been experimented. The novel trans-umbilical venous approach [53,54] will be reviewed in this section.

**Figure 26:**
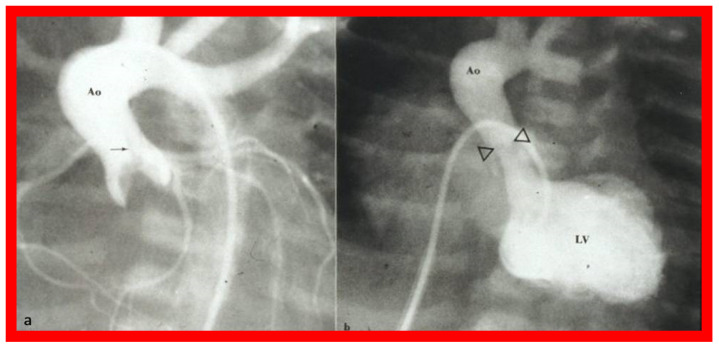
(**a**). Selected frame from the ascending aorta (Ao) cine-angiogram prior to balloon aortic valvuloplasty showing a domed aortic valve and a very narrow jet (arrow) of un-opacified blood from the left ventricle (LV) to the Ao. Post-stenotic dilatation of the Ao is also seen. (**b**). LV cine-angiographic frame following balloon valvuloplasty demonstrating dilated LV and wide jet of contrast material (arrowheads) across the aortic valve. Reproduced from Reference [54].

The author made it a practice to urge the neonatologists to insert an umbilical venous (UV) catheter once a cardiac baby is detected and place the tip of the UV catheter in the right atrium before the anticipated closure of the ductus venosus. During BAV procedure, the UV catheter is switched over a guidewire with a 5-F sheath with the tip of the sheath placed in the low right atrium [53,54,55]. Standard hemodynamic data are recorded, aortogram (Figure 26a) and/or left ventricular (LV) cine-angiogram secured, and the diameter of the aortic valve annulus is measured in multiple projections. Such data supplements echo-measured valve annulus diameter.

**Figure 27:**
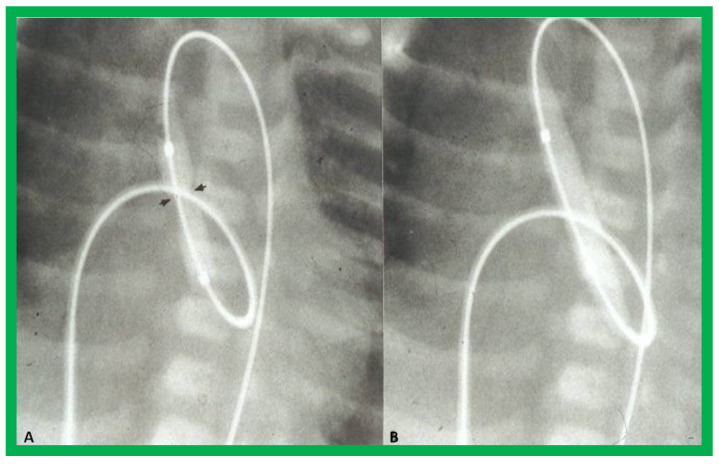
Selected cine-radiographic frames demonstrating the position of the balloon across the aortic valve, introduced anterogradely. Note the waisting (arrows) of the balloon during the initial phases of balloon inflation (**A**), which was completely abolished after full inflation of the balloon (**B**). Reproduced from Reference [54].

**Figure 28:**
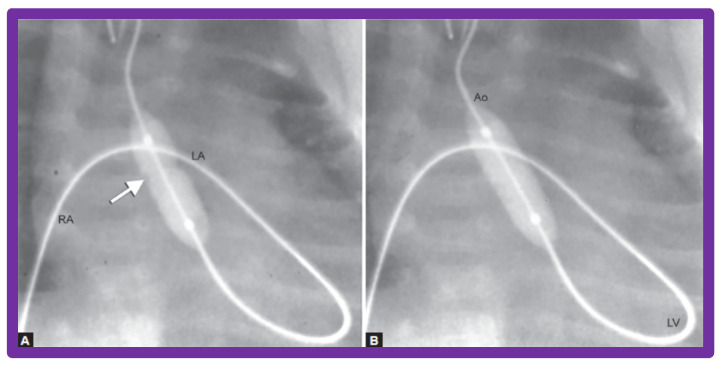
Selected cine frames demonstrating the position of the balloon across the aortic valve introduced anterogradely from the umbilical vein, right atrium (RA), left atrium (LA), left ventricle (LV), and aorta (Ao). (**A**) Note the waist (arrow) of the balloon which was completely abolished after further inflation of the balloon (**B**). Reproduced from Reference [55].

A #4-F multipurpose catheter (Cordis) or a similar catheter is placed in the UV sheath and pushed forward into the left atrium via the PFO and then through the mitral valve into the LV. With the help of a J-shaped and/or a straight, soft-tipped 0.035” Benston guide wires (Cook), the catheter is positioned in the ascending aorta and if possible, the tip of the catheter is negotiated into the proximal descending aorta. At this point, the guidewire is switched with a 0.018” or 0.021” J-tipped guidewire, suitable to accept the chosen balloon valvuloplasty catheter. The multipurpose catheter is withdrawn and a 6 to 8 mm diameter Tyshak II (Braun) or ultrathin (Meditech) balloon dilatation catheter is threaded over the guidewire from the UV, inferior vena cava, right atrium, left atrium, LV, and aorta. During this process, a wide loop of the guidewire in the LV should be maintained. The diameter of the balloon used for BAV should be 0.8 to 1.0 times the aortic valve annulus. Following placement of the balloon catheter across the aortic valve, the balloon is inflated with diluted contrast material with inflation pressures going up to the manufacturer’s recommendations, or till the waist of the balloon is abolished (Figure 27 and Figure 28). One or two more balloon inflations are performed to ensure adequate BAV. 

The balloon valvuloplasty catheter is switched over to a #4-F multipurpose catheter and the guidewire is withdrawn. Pullback pressure recordings across the aortic valve are documented and aortic root angiogram is performed. LV cine-angiogram (Figure 26b) may be performed as deemed suitable. Heparin is given at the start of the BAV and activated clotting times (ACTs) checked. Vancomycin is administered for prophylaxis because of extensive manipulation of the umbilical region during the BAV [53,54,55].

#### 3.2.1. Additional Procedural Details

**Figure 29:**
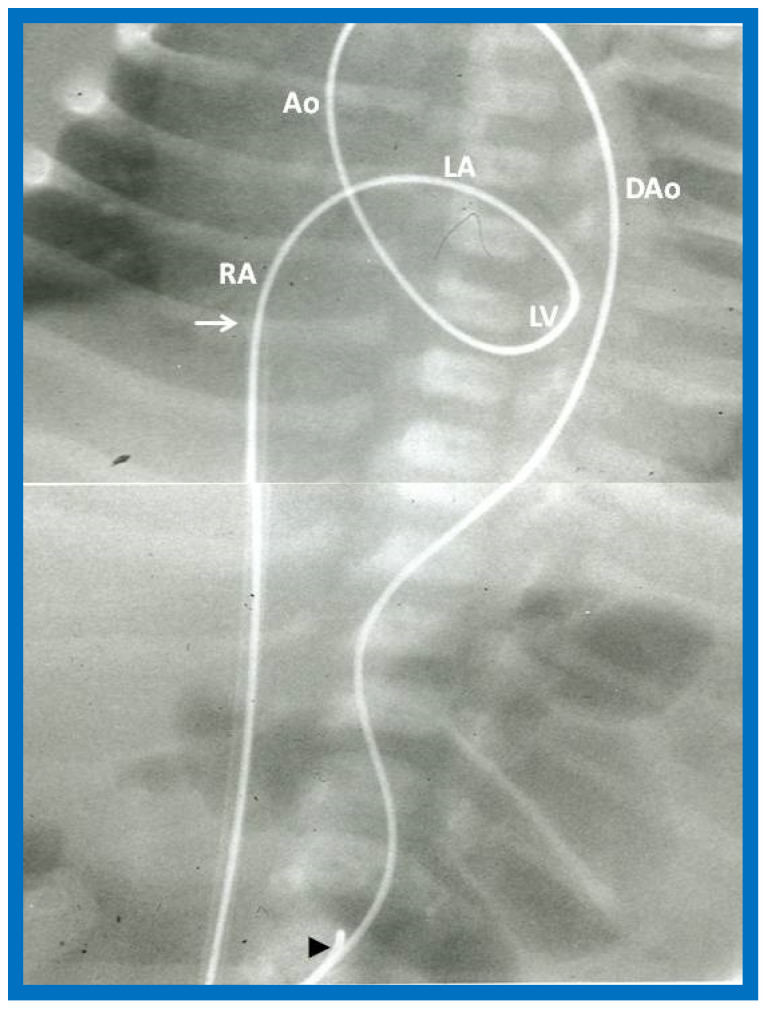
The course of the guide wire ‘‘rail’’ from the umbilical vein-to-umbilical artery for positioning the catheter across the aortic valve is demonstrated. The filled arrowhead shows the tip of the snare holding the wire. The tip of the umbilical venous sheath (arrow) is also shown. The wire ‘‘rail’’ courses through the right atrium (RA), left atrium (LA), left ventricle (LV), ascending aorta (Ao), and descending aorta (DAo). Reproduced from Reference [53].

In babies in whom the guidewire cannot be positioned in the descending aorta or the BAV catheter cannot be placed across the aortic valve, a gooseneck micro-snare (Microvena, White Bear Lake, MN, USA) may be sited in the descending aorta either via the femoral or umbilical artery. Then the tip of the anterogradely placed guide wire is snared and pulled down into the descending aorta and held in place. Thus, an umbilical venous-to-umbilical/femoral arterial wire “rail” is created (Figure 29). A mild traction on the descending aortic section of the wire rail facilitates placement of the BAV catheter across the aortic valve. It should be remembered that a wide wire loop in the LV should be maintained during this process. After the BAV procedure is finished, the guidewire is let go from the snare and removed. To prevent injury of the intracardiac structures, a catheter is maintained over the entire course of the guidewire during guidewire withdrawal [53].

**Figure 30:**
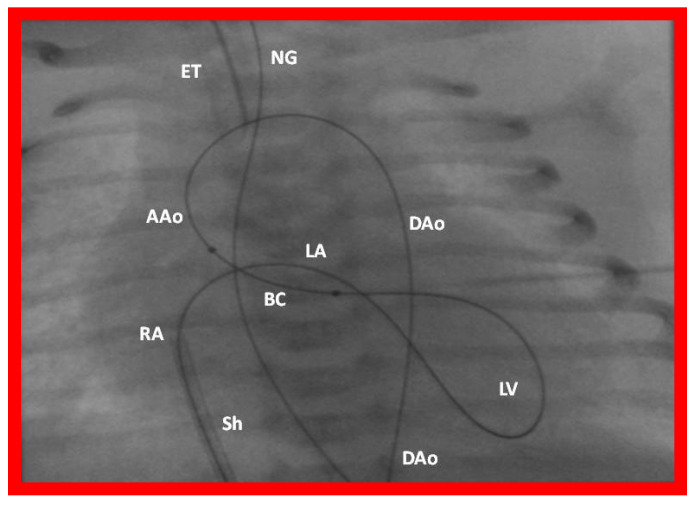
Selected cine-radiographic frame demonstrating the course of the guide wire from the umbilical vein (not shown) to the right atrium (RA), left atrium (LA), left ventricle (LV), ascending aorta (AAo), and descending aorta (DAo). The balloon catheter (BC) is positioned across the aortic valve without the use of a snare as shown in Figure 29 because of easy tack-ability of the Tyshak II catheter used in this case. The sheath (Sh) is seen in the RA, positioned via the umbilical vein. ET, endo-tracheal tube; NG, naso-gastric tube.

Subsequently, we have improved the technique with the use regular 0.021″ guide wires (Cook, Bloomington, IN) instead of extra-stiff Amplatz wires (Cook) and Tyshak-II catheters (Braun, Bethlehem, PA) (Figure 30) instead ultrathin balloon valvuoloplasty catheters (Meditech, Natick, MA) [54,56]. Since the use of these changes, it was not necessary to use snare nor to institute a guide wire loop. We also observed less arrhythmia during the BAV procedure, apparently because of employing less stiffer wires and better tracking of the Tyshak-II catheters [54,56].

#### 3.2.2. Comments

The concept of anterograde transvenous approach described by Hausdorf [51] and O’Laughlin [52] and their colleagues was adopted by us; we used the UV instead of femoral vein [53]. We initially thought that creating a guide wire rail (Figure 29) was necessary [53]; however, subsequent experience with the procedure [54,56] demonstrated that such guidewire rail is not necessary, especially in view of availability of less stiff guidewires and more trackable balloon catheters (Figure 30). 

Following the initial description of this procedure and successful results in one patient [53], we employed the technique in five more patients [54,56]. The trans-umbilical venous BAV procedure was successful in 80% (4 out of 5) patients. There was excellent relief of aortic valve obstruction in all four patients. The sole infant in whom we were unable to complete the BAV was secondary to a very small LV which did not allow the guidewire to be negotiated across the aortic valve. The experience gained in these studies [54,56] resulted in modification of the techniques as alluded to above (Figure 30).

Based on these results [53,54,56], we recommended trans-umbilical venous anterograde route for BAV in the neonate as an alternative option to other routes mentioned previously. Clearly, success of UV route of entry for BAV requires patency of the ductus venosus and the presence of a PFO [53,54,56].

In summary, anterograde trans-UV route for accomplishing BAV in newborn infants is achievable and is an effective substitute to retrograde femoral, carotid, or umbilical arterial and trans-femoral venous anterograde techniques [53,54,56]. The described results provide support to our advocacy and suggestion to utilize of the trans-UV anterograde approach as first option in the transcatheter treatment of critical AS in the newborn [54,54,56].

## 4. Aortic Coarctation

In this part aortic remodeling following BA of both native and post-surgical coarctation and biophysical response after BA of AC will be reviewed.

### 4.1. Aortic Remodeling

#### 4.1.1. Native Aortic Coarctation

**Figure 31:**
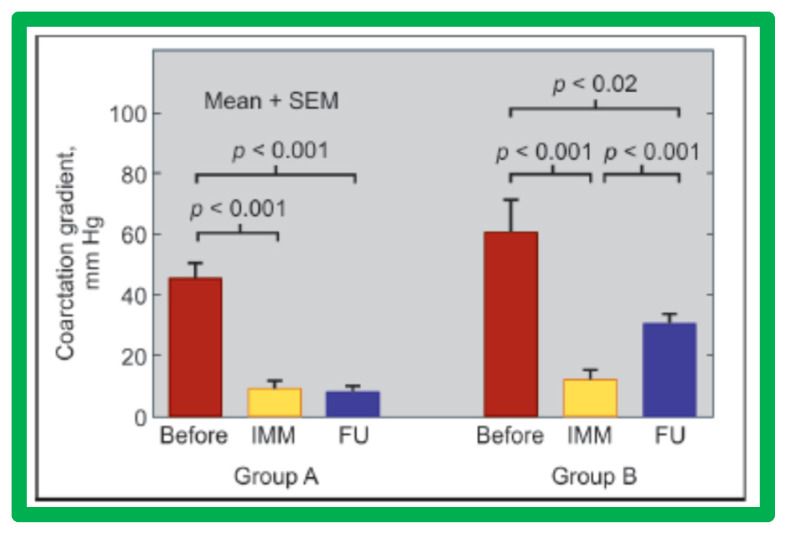
Bar graph showing immediate (IMM) and follow-up (FU) results of balloon angioplasty in Group A with good results (left panel) and in Group B with poor results (right panel). In Group A with good results, the coarctation gradients decreased significantly (*p* < 0.001) immediately after balloon angioplasty and remained low (*p* < 0.001) at follow-up. In Group B with poor results, the coarctation gradient also fell (*p* < 0.001) immediately after angioplasty but increased significantly (*p* < 0.001) at follow-up. SEM, standard error of mean. Modified from Reference [58].

We have undertaken a study to evaluate whether remodeling of the aorta occurs following successful BA of native aortic coarctation [57]. The study subjects are the same group of 30 children in whom we examined the causes of re-coarctation following balloon angioplasty of AC [58]. Based on the results of 6-to-30-month follow-up catheterization and angiographic data in 20 children, the patients were divided into two groups (Figure 31): Group A with good results (13 patients) and Group B with poor result (7 patients) [58]. 

**Figure 32:**
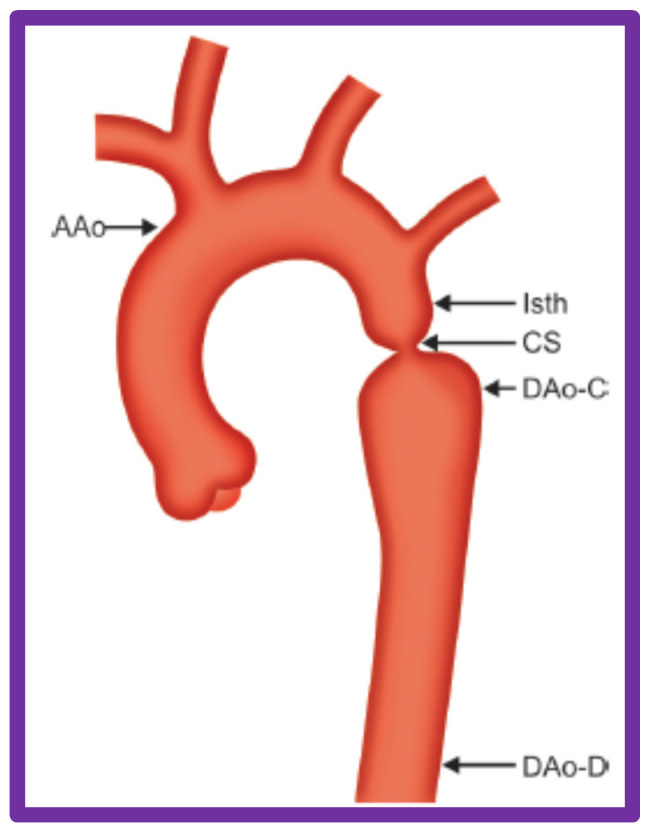
Diagram shows measurements of the aorta at five sites, namely, the ascending aorta proximal to the origin of the right innominate artery (AAo), isthmus (Isth), coarcted aortic segment (CS), and descending aorta distal to the coarctation (DAo-C) and at the level of the diaphragm (DAo-D) that were made on the angiograms performed prior to and at follow-up to examine if remodeling of aorta has occurred. The measurements were made in two angiographic views, corrected for magnification, and averaged. Modified from Reference [57].

**Figure 33:**
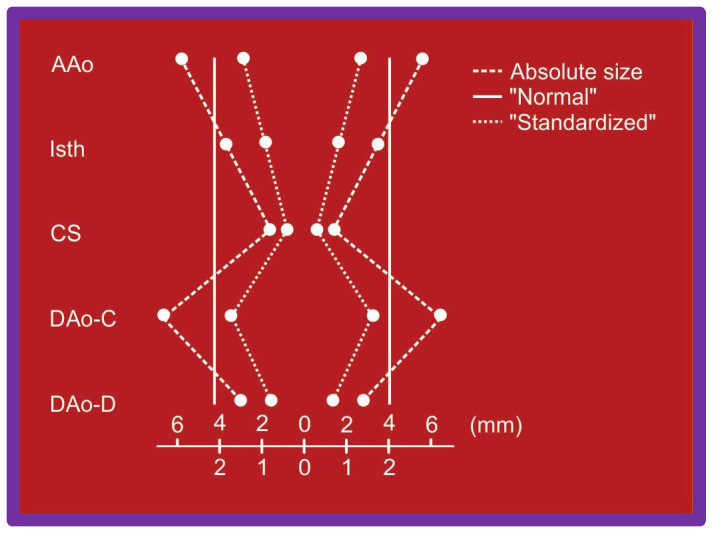
Diagram shows how standardized diameters of the aorta at the five locations were calculated for each case before angioplasty and at follow-up study. Abbreviations are same as those used in Figure 32. The variance of the diameter from normal was then calculated (not shown). Modified from Reference [57].

The ascending aorta, isthmus, coarcted segment and descending aorta distal to the coarctation and at the level of the diaphragm (Figure 32) were measured in two angiographic views and averaged after correcting for magnification. A standardized diameter [57] of the aorta at the five sites was derived (Figure 33) for each of the subjects separately both prior to BA and at follow-up. The variance of the diameter was then calculated. 

**Figure 34:**
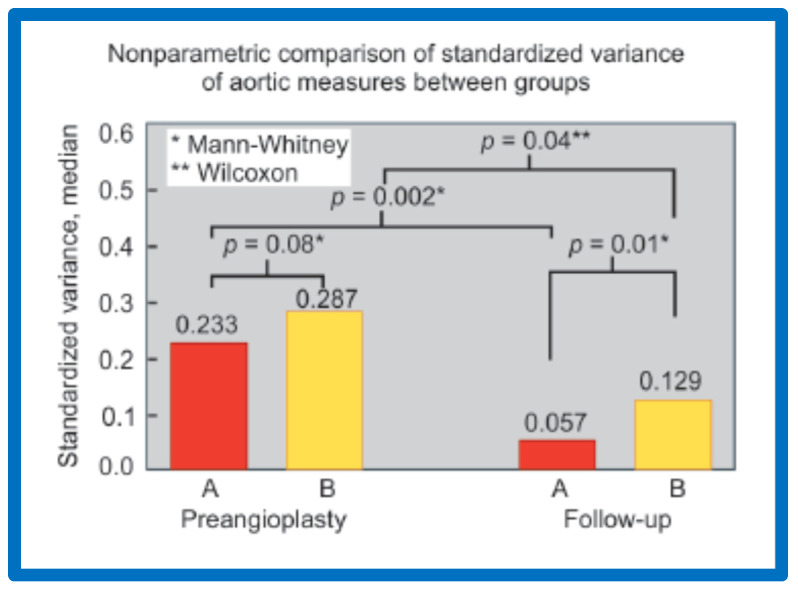
Bar graph shows comparison of the variances of standardized aortic diameters between groups A and B. The variance was similar (0.233 vs. 0.287; *p* > 0.05) in both groups before angioplasty (Pre-angioplasty). However, at follow-up the variances were different (0.057 vs. 0.129; *p* = 0.01). There was also a greater percent improvement at follow-up study (0.233 vs. 0.057; *p* = 0.002) in the group A with good results than in the group B with fair or poor results (0.287 vs. 0.129; *p* = 0.04). In the insert at the left upper corner, the type of nonparametric test used for comparison is denoted. Modified from Reference [57].

The variance of standardized aortic measurements (0.233 vs. 0.287) was similar (*p* > 0.05) in both groups prior to angioplasty (Figure 34). However, at follow-up, these measures (0.057 vs. 0.129) were different (*p* = 0.01); there was a greater percent improvement at follow-up study (0.233 vs. 0.057) in the group with good results than in the group with poor results (Figure 34). 

**Figure 35:**
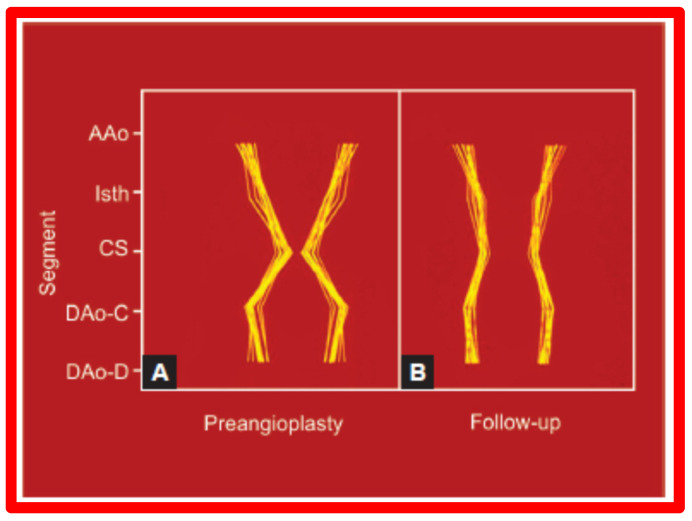
Schematic diagram of (**A**) standardized aortic diameters pre-angioplasty and (**B**) at follow-up in group A. Note improvement in that there is more uniformity of the various diameters of the aorta. Abbreviations are same as those used in Figure 32 and Figure 33. Modified from Reference [57].

**Figure 36:**
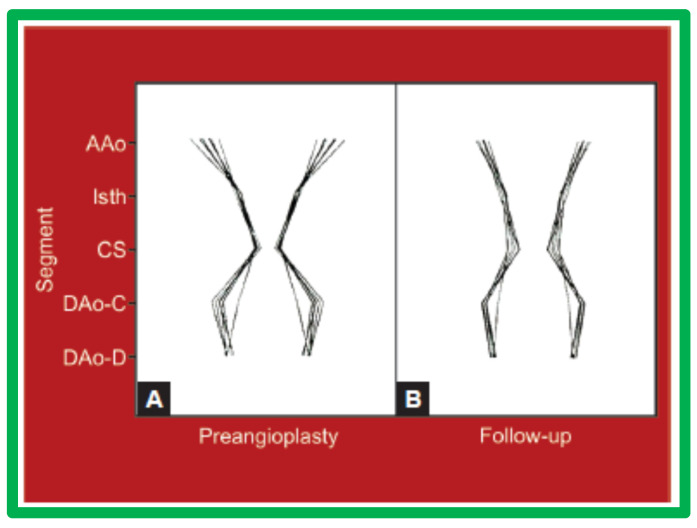
Schematic diagram of standardized aortic diameters pre-angioplasty (**A**) and at follow-up (**B**) in group B. Note no significant improvement in the diameters of the aorta. Abbreviations are same as those used in Figure 32 and Figure 33. Modified from Reference [57].

Line drawings of these data visually depict these changes in Figure 35 and Figure 36.

The rearrangement of the proportions of the aortic segments, as we named “remodeling”, was truly remarkable as shown by a pronounced reduction in the variation in the standardized aortic segment measurements in the good results group (Group A) as illustrated in Table 3 of our paper [57] and in Figure 35. By contrast, in Group B with poor or fair results, there was no improvement (Table 3 of our paper [57] and Figure 36). The remodeling of the aortic segments is likely be related to improved blood flow across this region, as has been demonstrated in fetuses and neonates [59]. This level of remodeling in this study was at follow-up duration of one year and we assume that there may be even better remodeling and normalization of the aortic segments at a longer follow-up duration. Based on these data, it was concluded that greater remodeling of the aorta takes place following effective BA of AC and such positive effect is likely to be related to normalization of flow across the opened aortic segments [57].

Surez de Lezo and his colleagues [60] approached this issue in a slightly different manner. They examined configuration angle between proximal and distal aortic segments and found that this angle increased from 169 ± 17° to 186 ± 17°; *p* < 0.05 at 10 ± 2 months after BA. They interpreted these findings to represent flow-dependent alignment of the aortic segments with greater alignment of the proximal with the distal aorta following BA [60].

Thus, the data from our study [57] as well as those of Surez de Lezo [60] would imply that the aorta assumes a more uniform appearance following successful BA.

#### 4.1.2. Post-Surgical Re-Coarctation

While we have not performed detailed remodeling studies for post-surgical re-coarctation such as those for native AC, we documented improvement in the diameter of the transverse aortic arch/aortic isthmus from 7 ± 3 mm to 10 ± 3 mm (*p* < 0.01) at follow-up [61], signifying aortic remodeling such as that described for native AC [57,60].

### 4.2. Biophysical Response of Coarcted Aortic Segment to Balloon Angioplasty

To further investigate the issues related to causation of re-coarctation after BA of native AC and potential role of elastic properties of the coarcted segment in re-coarctation, we have examined the biophysical response of the coarcted segment to BA [62].

**Figure 37:**
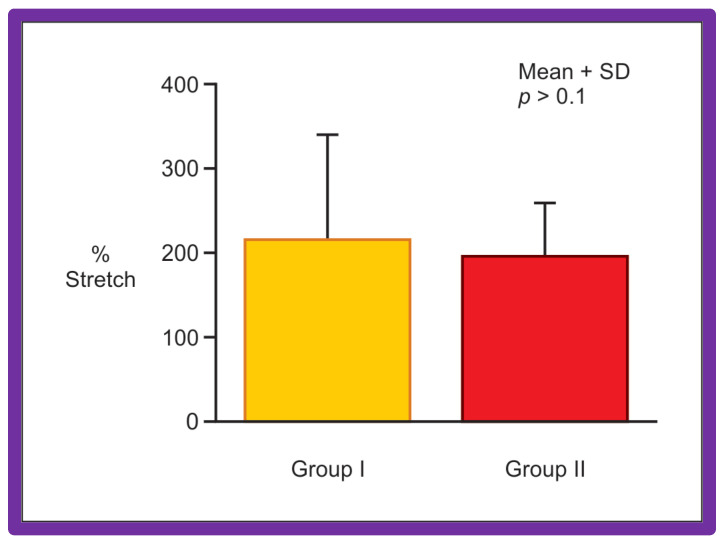
Bar graph shows comparison of the stretch applied during balloon angioplasty in group I with good results and group II with poor results. Note that similar (*p* > 0.1) stretch was applied in both groups. Mean + SD (standard deviation) is shown. Modified from Reference [63].

**Figure 38:**
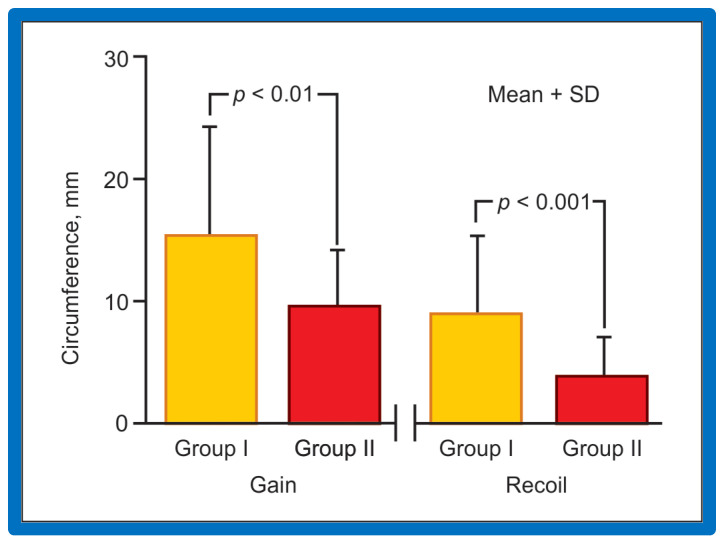
The bar graph shows comparison of gain and recoil after balloon angioplasty in group I with good results and group II with poor results. Note that both gain and recoil were higher (*p* < 0.01 to 0.001) in group I than in group II. Mean + SD (standard deviation) is shown. Modified from Reference [63].

**Figure 39:**
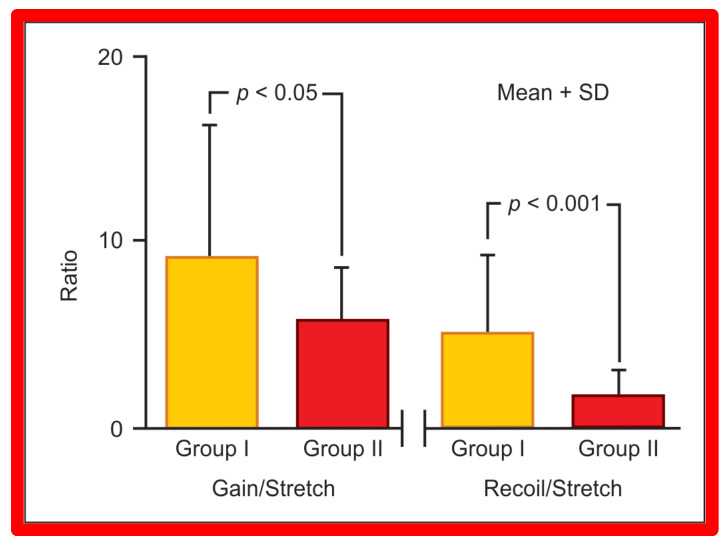
The bar graph shows comparison of gain and recoil, normalized to stretch in group I with good results and group II with poor results. All study subjects are included in this comparison. Note that both gain and recoil continue to be higher (*p* < 0.05–0.001) in group I than in group II. Mean + SD (standard deviation) is shown. Modified from Reference [62].

Data of 67 consecutive infants and children undergoing BA of native AC during an 8.7-year period ending September 1993 were examined [62]. Stretch (balloon circumference minus pre-balloon coarcted segment circumference ÷ pre-balloon coarcted segment circumference), gain (post-balloon coarcted segment circumference minus pre-balloon coarcted segment circumference), and recoil (balloon circumference minus post-balloon coarcted segment circumference) were derived from measurements acquired from cine-angiograms performed before and immediately after BA. At a median of 12-month follow-up, 15 (25%) of 59 children developed re-coarctation (gradient ≥ 20 mm Hg). Data on 44 patients in Group I without re-coarctation were compared with those of 15 patients in Group II with re-coarctation. The stretch (Figure 37) in Group I (218 ± 123%) was similar (*p* > 0.1) to that in Group II (190 ± 65%), indicating that similar balloon dilating stretch was exerted in both groups. The uncorrected gain and recoil were higher (*p* < 0.01 to 0.001) in Group I than in Group II (Figure 38). The gain (8.8 ± 8.0 vs. 5.7 ± 2.7 mm; *p* < 0.05) and recoil (5.1 ± 4.3 vs. 2.1 ± 1.1; *p* < 0.001), normalized to stretch remained larger in Group I than in Group II (Figure 39). 

**Figure 40:**
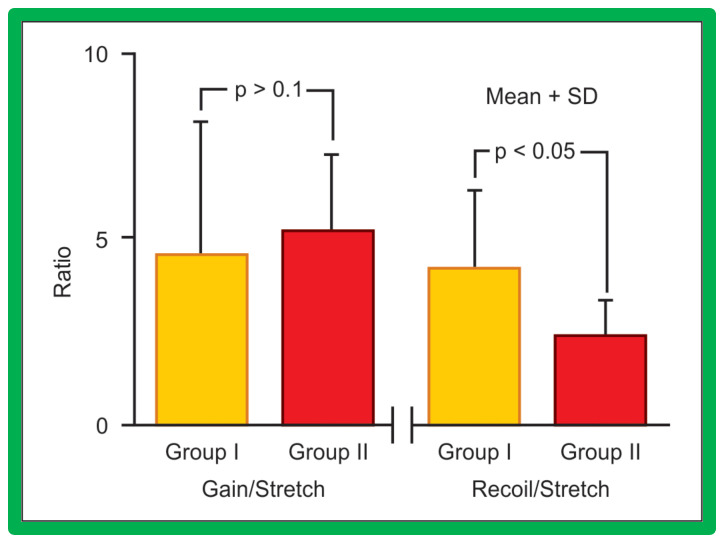
The bar graph shows comparison of gain and recoil, normalized to stretch in group I with good results and group II with poor results. Only infants are included in this comparison to exclude the influence of age and weight. Note that gain, normalized to stretch is similar (*p* > 0.1) but, recoil, normalized to stretch continues to be higher (*p* < 0.05) in group I than in group II. Mean + SD (standard deviation) is shown. Modified from Reference [62].

However, the Group I patients were older than Group II patients (64.3 ± 53.4 months vs. 16.8 ± 31.1 months; *p* < 0.001). Similarly, the weights (20.6 ± 14.8 kg vs. 7.6 ± 6.4 kg; *p* < 0.001) were different. Consequently, it is possible that the age and weight of the patients may account for the differences in the findings described above. Therefore, we examined the data of only infants (≤12 months). The stretch (196 ± 10 vs. 185 ± 56; *p* > 0.1) and gain normalized to stretch (4.6 ± 2.1 vs. 5.1 ± 2.1; *p* > 0.1) were similar, but the recoil was better (4.1 ± 2.2 vs. 2.2 ± 0.9; *p* < 0.05) (Figure 40) in Group I without re-coarctation than in Group II with re-coarctation. 

Better recoil in the patients without re-coarctation infers preservation of intact elastic tissue in the coarcted segment [62]. The elastic properties [64,65,66] may not have been preserved in the re-coarctation group with less recoil; this may have caused re-coarctation. There might be a more severe amount of cystic medial necrosis [64,65,66] in the re-coarctation group than in the no re-coarctation group. However, this needs confirmation in future studies.

## 5. Summary and Conclusions

While studying the results of balloon dilatation techniques in subjects with congenital stenotic lesions of the heart, several parallel observations were documented. In this review, the observations/phenomena/innovations associated with balloon dilatation of PS, AS, and AC were discussed. RV infundibular gradients occur following BPV in nearly 30% of children; these are more frequent with increasing severity of PS and advancing age of the patient. There is a tendency for spontaneous resolution of infundibular stenosis. Infundibular gradients that are more than 50 mmHg are benefited by beta-blocker therapy with occasional need for surgical intervention. Cardiologists should not be dissuaded from performing BPV because of development infundibular obstruction. Our investigation determined that ECG gets better following successful BPV, and the ECG is a helpful adjunct in the assessment of results of BPV. ECG proof of improved gradient across the pulmonary valve does not become evident until six months after BPV. While Doppler data are not sufficiently sensitive to detect improved RV filling after BPV, increased systemic arterial saturation and decreased right-to-left shunt across PFO suggest improved RV filling and increased RV compliance. Detailed examination of influence of B/A ratios on the results of BPV indicate that B/A ratios of 1.2 to 1.25 are likely to produce effective relief of PS while at the same time may help in preventing significant PI at long-term follow-up. Comparison of double-balloon technique with single balloon BPV revealed similar results with no clear advantage for double-balloon technique. Investigation to determine causes of AI following BAV indicated immediate post-BAV AI by Doppler is predictive of late AI, but the true cause is not known. It is likely to be a mixture of poor valve morphology and use of large-sized balloons. Anterograde trans-UV route for performing BAV in neonates was described which avoids injury to the femoral arteries. Such a procedure was successful in 80% of patients. The author recommends use of trans-UV anterograde approach as first option in the transcatheter treatment of critical AS in the neonate. Aortic remodeling takes place after successful BA of both native and post-surgical ACs and is presumably due to restoration of normal blood flow across the coarcted aortic segments. Finally, examination of biophysical properties of coarcted aortic segment showed that while the stretch applied and gain achieved by BA was similar in both good and poor results groups, the recoil was poorer in the group with re-coarctation than those without. This may imply lack of preservation of elastic properties in subjects who had re-coarctation. The described observations/phenomena/innovations add to a better understanding of balloon dilatation of stenotic lesions of the heart.

## Figures and Tables

**Table 1:** Management of Infundibular Obstruction.

1. Consider the possibility of development of infundibular obstruction after BPV in all patients with severe valvar PS
2. Perform careful pressure pullback recordings across the pulmonic valve and RV outflow tract both prior to and 15 min after BPV *.
3. Perform RV cine-angiography and scrutinize these angiograms for infundibular obstruction both before and 15 min after valvuloplasty *.
4. Use a valvuloplasty balloon that will result in a B/A ratio of 1.2 to 1.25 [9,10].
5. Use a double-balloon technique when the pulmonary valve annulus is too large to dilate with a commercially available single balloon. When a double balloon technique is used, the effective diameter of both balloons together should be used for calculation of the B/A ratio. Effective balloon diameter may be calculated by the formula: 0.82(D1 + D2) [11,12].
6. If pulmonary valve dysplasia is present, a B/A ratio as high as 1.5 may be necessary for effective relief of pulmonary valve obstruction [13].
7. Balloons larger than 1.5 times the size of the pulmonary valve annulus should not be used because such large balloons may damage the right ventricular outflow tract muscle [14]. In addition, balloons more than 1.5 times the size of the pulmonary valve annulus did not produce better immediate or intermediate-term results when compared with the subgroup in whom a B/A ratio of 1.2 to 1.4 was achieved during balloon valvuloplasty [15,16] and the extra-large balloons may precipitate an infundibular reaction.
8. If angiographic (Figure 2A), pressure (Figure 1) and/or echo-Doppler (Figure 3B) data suggest, significant residual infundibular obstruction, beta blocker drug therapy may be necessary; we recommend it if the residual gradient is more than 50 mmHg [2,6].
9. If results of follow-up echo-Doppler or catheterization and angiographic studies performed 6 months to 1 year after balloon valvuloplasty show residual infundibular gradients ≥ 50 mm Hg, then surgical resection of the infundibular muscle may be considered. If there is significant residual valvar obstruction, repeat BPV with adequately sized balloon(s) would be our therapeutic choice [8].

* Some cardiologists use echo-Doppler evaluation instead. Modified from Reference [8].

**Table 2:** Frequency of repeat balloon and dilatation significant residual gradients in various subgroups.

	Number of Patients Needing Repeat BPV	Number of Patients with Gradient > 30 mmHg
Group I (B/A ratio < 1.0)	4	6
Subgroup IIA (B/A ratio of 1.01 to 1.2)	1	2
Subgroup IIB (B/A ratio of 1.21 to 1.4)	0	0
Subgroup IIC (B/A ratio > 1.41)	0	0

BPV, balloon pulmonary valvuloplasty; B/A ratio, balloon/annulus ratio. Modified from Reference [16].

## Data Availability

Not applicable.
